# Social dominance orientation underlies social valuation in a competitive social hierarchy

**DOI:** 10.3389/fpsyg.2025.1615364

**Published:** 2025-09-15

**Authors:** Zhang Qiang, Masahiko Haruno

**Affiliations:** ^1^Center for Information and Neural Networks, National Institute of Information and Communications Technology, Osaka, Japan; ^2^Graduate School of Frontier Biosciences, Osaka University, Osaka, Japan

**Keywords:** social dominance orientation, fairness, social hierarchy, dominance, inequity aversion, social valuation, wellbeing

## Abstract

**Introduction:**

Humans are constantly evaluating whether they are in a fair situation. However, perceptions of fairness vary widely across individuals. What governs this individual variability in fairness perception remains poorly understood. In this study, we hypothesized that individual sensitivity to social dominance hierarchies influences fairness perception and therefore tested whether social dominance orientation (SDO) accounts for such individual preferences.

**Method:**

We first assessed SDO scores in 29 participants using a standard SDO questionnaire. Participants then completed two competitive tasks. In the first task, they engaged in repeated competitions to establish a social hierarchy among opponents. In the second (main) task, participants played against opponents whose ranks were determined in the first task. Each trial in the second task awarded both a performance-based reward and a variable bonus (independent of win/loss) to both the participant and their opponent. Participants rated the desirability (social valuation) of each bonus distribution on a 4-point scale (1 = least preferable, 4 = most preferable). Using LASSO regression, we examined how self-reward, bonus inequity, outcomes (win/loss), opponent rank, SDO, and their interactions affected social valuation.

**Result:**

We found that the outcome modulated the influence of bonus inequity on social valuation, with inequity having a stronger effect on win trials. Crucially, low-SDO individuals tended to favor larger bonuses for winners in loss trials, whereas high-SDO individuals consistently preferred larger bonuses for themselves regardless of the outcome. Moreover, high-SDO participants rated bonuses more favorably when facing lower-ranked opponents, while low-SDO individuals showed the opposite pattern.

**Discussion:**

These findings suggest that SDO, interacting with the competitive outcome, plays a key role in shaping dynamic social valuation.

## 1 Introduction

In our daily lives, we consistently assess whether society treats us fairly. Imagine working at a company where both your performance and that of your colleagues are transparent, and you receive a monthly bonus, which may or may not reflect your performance precisely. While the absolute amount of your bonus is undeniably important, your mind also considers other factors to calculate your social valuation, such as how your bonus compares to those of your colleagues, performance metrics for yourself and your peers, social rank of yourself and your colleagues, and the interplay among those factors. We are happy when we feel we are treated fairly, yet it remains poorly understood what exactly defines the process to reach this conclusion and how much individual difference exists in the process.

Social hierarchy is observed in a broad range of animals (Kemmelmeier, 2005; Strauss et al., 2022) including humans (Qu et al., 2017), and social comparison based on the hierarchy is a well-perceived component of social valuation. In particular, the effects of social inequity (i.e., resource disadvantage and advantage) have been intensively studied. These studies demonstrated that people dislike inequity and often make decisions to avoid it. Specifically, when people are asked to rate or choose a money distribution between the self and other, they often prioritize the equity between the two (Fehr and Schmidt, 1999; Van Lange, 1999; Fliessbach et al., 2007; Tricomi et al., 2009; Haruno and Frith, 2010). However, most of these studies did not consider contexts other than money distribution between the self and other(s). Only a single study examined how other contextual information alters the effects of social comparison on social valuation (Wright et al., 2011) and reported that participants have different attitudes toward the same distribution. That is, participants felt it less acceptable if they received a money distribution from the group that always gave an unfair distribution, while they tended to accept the same money distribution if it was from the group that always gave a fair distribution.

Social position achieved by one's own action (e.g., position ranked by win or loss) also affects a person's social valuation. One report tested a rank-income hypothesis, according to which people gain utility from the ranked position of their income within a comparison group (Boyce et al., 2010). That report found that the ranked position of an individual's income predicts general life satisfaction, whereas absolute income and reference income have no effect. However, to quantify the balance between absolute income and the ranked position in social valuation, more qualitative experiments are needed. In addition, rank and outcome (i.e., win/loss) may have a correlated effect with each other.

Connected to rank, there are remarkable individual differences in attitudes toward social dominance and hierarchical societies. Those with a high preference for dominance often display traits considered self-centered, and they view exploitation as both natural and necessary (Bergh and Sidanius, 2021). Such inclinations can be quantified using the Social Dominance Orientation (SDO) questionnaire, a tool to gauge one's preference for hierarchical dominance (Pratto et al., 1994). While many studies on SDO have focused on perceptions of social dominance hierarchies, few have delved into how SDO affects social valuation. For instance, one related study indicated that individuals with high SDO feel a diminished social gap between themselves and upper-class groups (Wang et al., 2019). One recent study using a meta-analysis reported that SDO-7 is a reliable measure for assessing overall SDO (mean α ≈ 0.83; Berry, 2023). It was also reported that SDO has high consistency with other personality traits (Jackson and Gaertner, 2010), and a third large-scale study across countries illustrated that people's SDO can track their preference on inequity and violence (Kunst et al., 2017). Together, these studies suggest that SDO is a reliable and stable personality trait.

Considering these backgrounds, the aim of this study is to elucidate how social valuation is constructed by different individuals depending on their SDO, outcome (win or loss), rank, inequity and absolute income (reward) and the interactions among these factors. Unlike previous studies, we dissociate inequity and outcome in our competitive game task, where we allocated a range of bonuses to participants and their opponents in addition to outcome-dependent rewards. Participants were asked to evaluate bonus distribution, ranging from least favorable (1) to most favorable (4). With this information, we attempted to characterize participants' social valuation by contextual information including SDO, outcome and bonuses. We hypothesized that outcomes (win/loss) and individuals' SDO modulate the influence of social inequity: high SDO individuals show anti-social preference, such as being more satisfied with the unfair distribution. We tested this hypothesis quantitatively by using a LASSO regression on the social valuation data, which can provide reliable results for a relatively limited number of trials to the number of explanatory variables.

## 2 Method

### 2.1 Participants

In this study, 45 participants (21 females and 24 males) aged 18 to 27 years old (average = 21.5, SD = 2.05) were initially recruited. Ethical committees of the NICT approved this study, and all participants gave informed consent.

We used G-power software to estimate the number of participants necessary for achieving a reasonable statistical power (Version 3.1.9.7. https://www.psychologie.hhu.de/arbeitsgruppen/allgemeine-psychologie-und-arbeitspsychologie/gpower). Specifically, we adopted the bivariate model and set the parameter (two tails, α = 0.05, power = 0.95, correlation H1 = 0.6, correlation H0 = 0). The required sample size was estimated to be 30, suggesting that to reach a reasonable statistical power (0.95) for the effect size (i.e., correlation coefficients between β and SDO scores around 0.6), we need around 30 participants (Section 2.3.2).

### 2.2 Competitive game task

**Figure 1:**
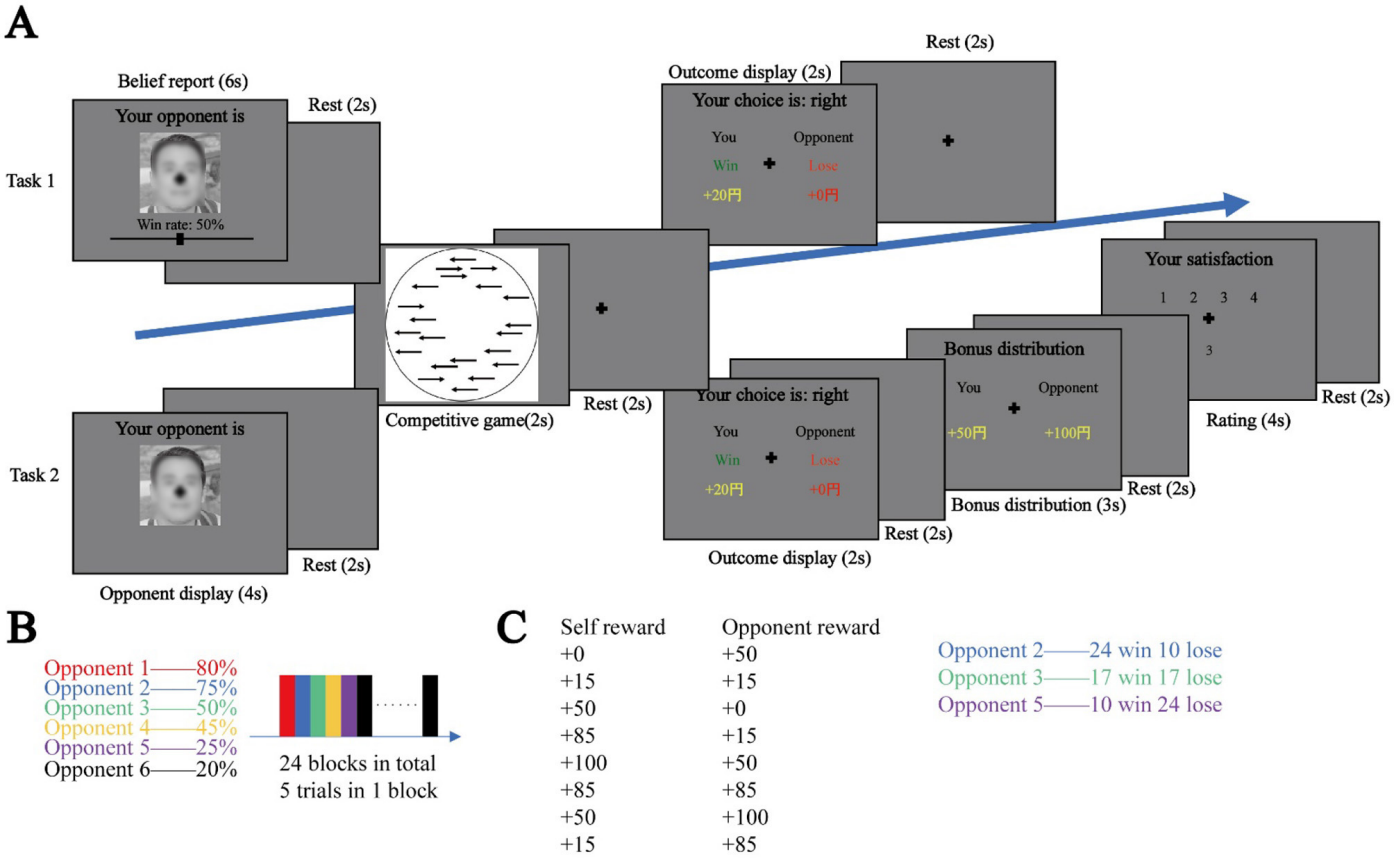
Experimental tasks and settings. **(A)** Task 1: learning task: participants in each trial report their expectation of winning against the opponent appearing on the screen. After a break of 1.5 s, they play a competitive game. At the end of the trial, they are informed of the total reward they are to receive. Task 2: social valuation task: participants in each trial first see the opponent they are to play against. After a break of 1.5 s, they play the same competitive game as in Task 1. At the end, they are informed of the outcome. The participants are then provided a bonus distribution, which they rate from 1 to 4 (a higher score means a more favorable distribution). **(B)** Participants will play against 6 different opponents, against each opponent, participants have different win rate. In task 1 there are 24 blocks, in each block, participants will play against with one opponent for 5 trials. **(C)** in task 2, there are 8 different possibilities of distribution, participants will play against 3 opponents which are from task 1. Face taken from Open AI.

The experiment consisted of two parts: the first was the learning task, and the second was the social valuation task. In the learning task (Task 1, Figure 1A), participants were shown their opponents in the beginning of each trial and asked to report their expectation of winning against the opponents by sliding a bar under the face picture. After a break of 1.5 s, the participants played a competitive game with the same opponents, in which 30 arrows appeared on the screen, and the participants pressed a button to indicate the direction to which the majority of the arrows are pointing.

The participants were informed that they were competing with their opponents and that the one who answered correctly faster would win the game (if both are incorrect, the slower one will win), but in fact the win rate against the opponent was pre-determined (Figure 1B).

After another break of 1.5 s, the participants saw the outcome of the game, and the winner received 20 yen and the loser nothing. This task included 20 trials for one opponent, and 5 trials against the same opponent constituted a block. After the participants finished one block, they moved to the next block to play against another opponent. One block for each of the 6 opponents appeared in one round, and there were 4 rounds in total.

After finishing the learning task, the participants ranked their 6 opponents. They next entered the social valuation task (Task 2 Figure 1A), where only 3 out of the 6 opponents appeared. Upon seeing an opponent's face, the participants played the competitive game and saw the outcome for 2 s. Next, a distribution of self-bonus (Bs) and opponent-bonus (Bo) appeared on the screen, and the participants were asked to rate the distribution from 1 (least favorable) to 4 (most favorable). The participants played against each of the opponents 34 times, and there were 102 trials in total. Eight distributions were used (Figure 1C). For opponent 2, these 8 distributions appeared 4 times, but the distribution of 85-85 and 85-15 both appeared one extra time in the lose situation, resulting in more equal and self-advantageous situations. For opponent 3, the 8 distributions appeared twice in both the win and lose situations, and the 85-85 distribution appeared one extra time in both situations so that we could observe more equal situations. For opponent 5, the 8 distributions appeared 4 times, but the 85-85 and 85-15 distributions both appeared one extra time in the win situation to obtain more information for equal and self-advantageous situations. After finishing the task, we interviewed the participants.

In addition to a base compensation of 4,000 yen, participants were informed that they would receive an outcome-based (game outcome and bonus) honorarium of −500 yen to +500 yen, but since the win rate was pre-determined before the experiment, their total reward was assigned to a value between 3,500 and 4,500 yen a priori.

After the experiment, we interviewed the participants. We confirmed that participants believed that the opponents' behaviors were produced by real individuals and then briefed them that the game grade (hierarchy) had been designed prior to the experiment. This level of reality was achieved by collecting the task behaviors of five people and taking their behaviors into account when we designed the opponents' behaviors. In addition, no participant reported changes in emotion during the experiment.

### 2.3 Data analysis

To analyze participants' social valuation (rating), we considered 5 factors (main effects): Bs, max (Bs-Bo,0), max (Bo-Bs,0), outcome (encode win as 1, lose as 0), and opponent group (encode opponent 5 as 3, opponent 3 as 2, opponent 2 as 1). All five factors and their interactions were standardized as z-scores and entered into the LASSO regression, since the number of these main variables and interactions was large for the number of participants and Lasso regression can automatically remove unimportant terms during regression and improve the explanation ability (see Section 2.3.1). The dependent variable in this LASSO regression analysis was the ratings in each trial.

The coefficient β for each term was estimated using a 10-fold cross validation, and the one with the minimum deviance (mean squared error, or MSE) was selected (see Section 2.3.1). We also conducted a Generalized Linear Model (GLM) analysis on the same data. To see the effects of the prediction error, we replaced the group variable with the prediction error to conduct another Lasso regression (Supplementary Figures S3, S4).

#### 2.3.1 Lasso regression

We considered multiple factors and their interactions, and the possibility that the contribution of these terms may differ across participants. To identify impactful terms for different subjects, we applied a Lasso regression. A Lasso regression is a method that conducts term selection and prediction. Lasso can avoid overfitting when many terms are included in the regression model (Santosa and Symes, 1986; Tibshirani, 1996).

For every participant, a Lasso regression is formalized by the following equation,


$$\begin{array}{l} y=\;\overset{p}{\underset{i=1}{\sum }}H_{i}\beta_{i}+\varepsilon , \end{array}$$

where $y={\left[y_{1},\ldots ,y_{n}\right]}^{T}$ is a vector consisting of the ratings in trials, n is the number of the trials, and p is the number of terms.

We defined *H* as the matrix of terms with p columns, where each column represents one term's value over n trials.


$$\begin{array}{l} H=\left[\begin{array}{ccc} x_{1}^{1}\; & \ldots \; & x_{1}^{p}\\ \vdots \; & \ddots \; & \vdots \\ x_{n}^{1}\; & \ldots \; & x_{n}^{p} \end{array}\right] \end{array}$$

$\beta ={\left[\beta_{1},\ldots ,\beta_{p}\right]}^{T}$ is the weight matrix and was calculated by solving


$$\begin{array}{l} \beta^{\prime }={\text{argmin}}_{\beta }||H\beta -y|{|}_{2}^{2}+\lambda ||\beta \;|{|}_{1}. \end{array}$$

**Figure 2:**
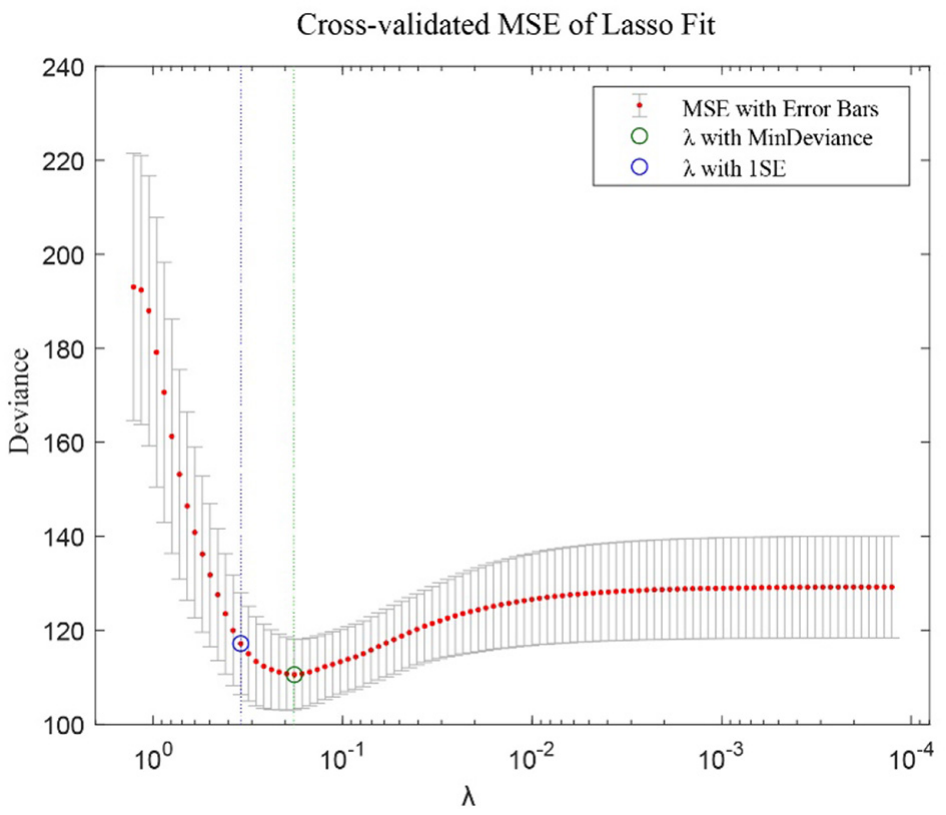
λ values and corresponding MSE (mean squared errors) in the LASSO regression. The plot exemplifies how the MSE changed when λ was altered. The optimal λ was defined as the point of the minimum MSE.

A higher λ penalizes larger β, such that the number of non-zero βs decreases. As shown in Figure 2, the LASSO algorithm gradually decreased λ and calculated the predictive MSE (by a 10-folds cross validation) for the various λs, and the optimal λ was selected at the lowest deviance (MSE) to guarantee generalizability. In LASSO, if a model does not generalize, all coefficients are estimated to be zero.

#### 2.3.2 Participant selection

We selected participants who successfully learned the dominance hierarchy in the learning task and analyzed their data in the social valuation task. Because three opponents (2, 3, 5) were used in the social valuation task, our criterion was that participants at least ranked these three opponents correctly at the end of the learning task.

Specifically, there should be at least a 10% difference between two opponents. If there was only a 5–10% difference, we checked the answers to the questionnaire given after the learning task. We used the participant only if the ordering answer was correct. Thirty-three participants learned the dominance hierarchy successfully.

We next checked the behavior data in the social valuation task. Consequently, 4 of the 33 participants were removed because of constantly pushing the same button, which suggested that they were not concentrating on the task. In the linear regression, we used *R*^2^>0.7 as the criterion to select the participants who are well-described by the linear model after consulting several studies (Ishigami and Homma, 1990; Vijayabanu and Arunkumar, 2018). As a result, 29 participants were used in the subsequent analysis.

As described in Section 2.1 our power analysis based on the G-power software suggested that the required number of participants was around 30. Thus, the 29 participants used in this study is likely sufficient for statistical analysis.

### 2.4 Social dominance orientation (SDO)

Social dominance orientation (SDO) is a personality measure of an individual's preference for hierarchy within any social system and the domination over lower-status groups. A widely used version of SDO is the SDO-7, which divides SDO into two subscales (Ho et al., 2015): one is SDO-D (Dominance), which describes the belief that some individuals should be in a more superior position, and the other is SDO-A (Anti-Egalitarianism), which describes the belief that a certain group of people ought to get more than other groups.

Since the first release of SDO, researchers have attempted to improve it, and with the most widely used current version including the 16 items shown below, each with 7 different scales. SDO was reported to show a correlation with beliefs such as that the world is a cruel jungle and that the fittest should survive (Chirumbolo et al., 2016).

#### 2.4.1 Dominance sub-scale

Some groups of people must be kept in their place.
It's probably a good thing that certain groups are at the top and other groups are at the bottom.
An ideal society requires some groups to be on top and others to be on the bottom.
Some groups of people are simply inferior to other groups.
Groups at the bottom are just as deserving as groups at the top (reverse-scored).
No one group should dominate in society (reverse-scored).
Groups at the bottom should not have to stay in their place (reverse-scored).
Group dominance is a poor principle (reverse-scored).

#### 2.4.2 Anti-egalitarianism sub-scale

We should not push for group equality.
We shouldn't try to guarantee that every group has the same quality of life.
It is unjust to try to make groups equal.
Group equality should not be our primary goal.
We should work to give all groups an equal chance to succeed (reverse-scored).
We should do what we can to equalize conditions for different groups (reverse-scored).
No matter how much effort it takes, we ought to strive to ensure that all groups have the same chance in life (reverse-scored).
Group equality should be our ideal (reverse-scored).

## 3 Results

**Figure 3:**
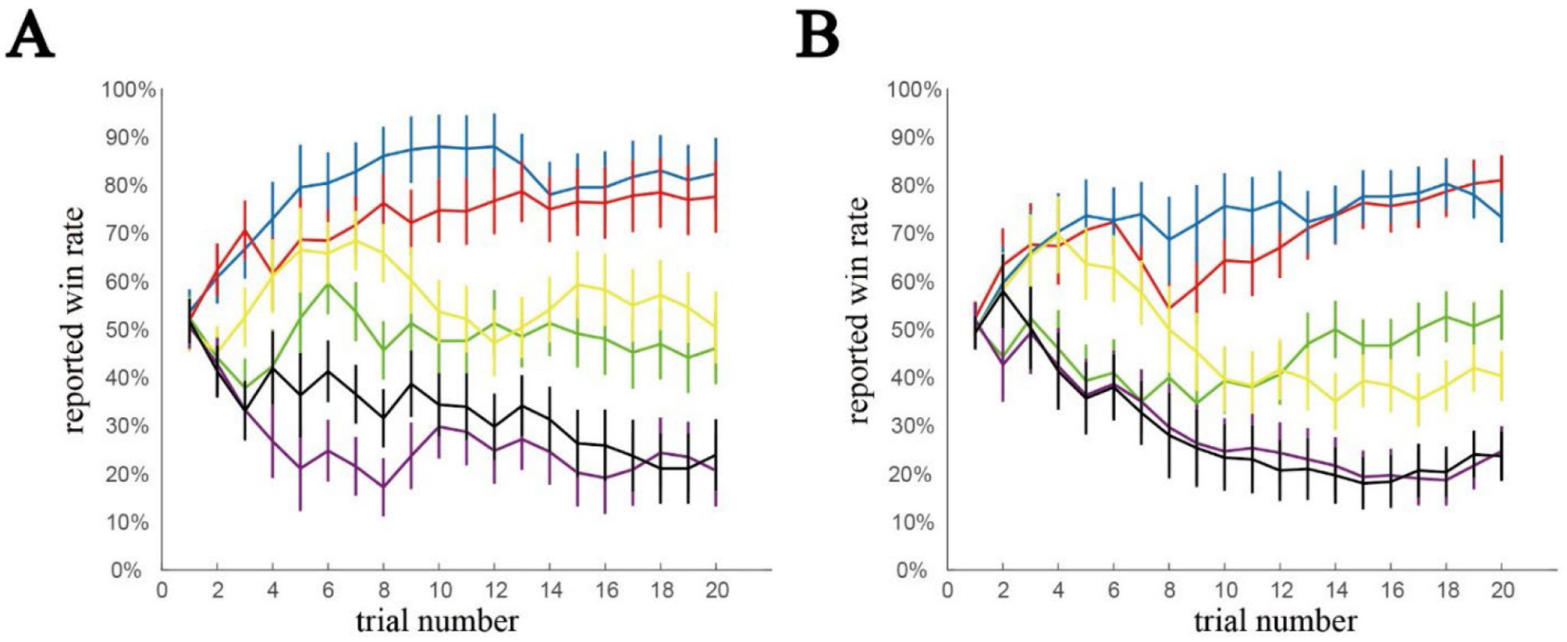
Reporting belief from well-learned participants in the learning task. The expected win rate determined by the participants. We prepared two patterns in task 1 to avoid the order effect: the number of win and loss trials are the same, but the sequence order was different. **(A)** Pattern 1. **(B)** Pattern 2. Error bars ±0.5*std. Irrespective of the pattern, the participants distinguished 3 different groups from 6 opponents.

In the learning task, we used two different sequences of opponent-outcome settings: pattern 1 with 23 participants, and pattern 2 with 22 participants: among them, 33 learnt the hierarchy correctly. We deemed these 33 as “well-studied.” The expectation of win reported by well-studied participants trial by trial is shown in Figure 3, with colors representing different opponents. These results confirm that the participants successfully learned the win probabilities of the opponents for the different learning sequences: pattern 1 (Figure 3A) and pattern 2 (Figure 3B).

Among the 33 participants who learnt the hierarchy correctly in the learning task, the behaviors of 29 participants in the social valuation task were reasonably explained by our linear model. Therefore, we analyzed the data of these participants in the subsequent analysis.

To identify the terms that influence the participants' social valuation, we conducted the LASSO regression (see **Methods**; data with the GLM is shown in Supplementary material) to handle many regressors with a limited number of participants. Specifically, we used self-bonus: Bs, advantage: max (Bs-Bo,0), disadvantage: max (Bo-Bs,0), outcome: (encode win as 1, lose as 0), group: (encode 1, 2, and 3), and their interactions as regressors.

**Figure 4:**
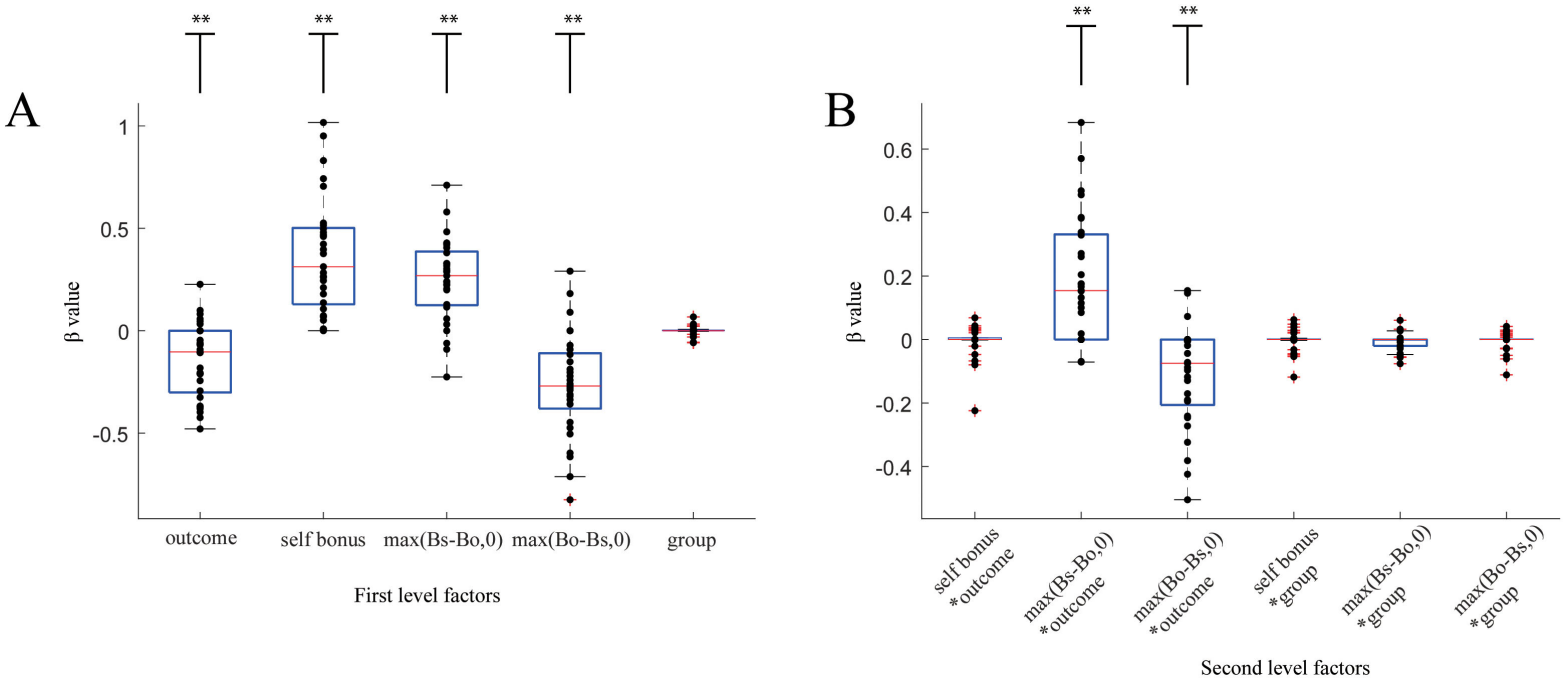
Boxplot of the LASSO β values for the 5 main effects **(A)** and their interaction terms **(B)**. **(A)** Five main effects were used in the LASSO regression. β values for the outcome, self-bonus, max (Bs-Bo,0), and max (Bo-Bs,0) were significantly different from 0, indicating that these terms strongly and widely impacted the participants' social evaluation. For the group, a smaller number of participants showed none-zero β values, indicating that a limited number of participants were influenced by this term. **(B)** Six second-level interaction terms were introduced into the LASSO regression. β values for max (Bs-Bo,0)*outcome and max (Bo-Bs,0)*outcome were significantly different from zero, illustrating that advantage and disadvantage influenced participants' social valuation by interacting with the outcome. **p < 0.01.

Figure 4 shows β (weight) values for the LASSO regression. We can see that β values for self-bonus and advantage are positive, while those for disadvantage are negative, suggesting the consistent importance of the three parameters across all participants (Figure 4A). Importantly, the sign of β for the outcome and group took both positive and negative values, indicating large individual differences in the effect of the outcome and group on social valuation. We also see that the outcome has a stronger effect on participants' social valuation in comparison than the group. Consistent with this, as shown in Figure 4B, β values for the interaction terms between the outcome and advantage and between the outcome and disadvantage exhibited significant positive and negative values, suggesting that the effects of advantage and disadvantage on social valuation depend on the outcome (i.e., win/loss).

**Figure 5:**
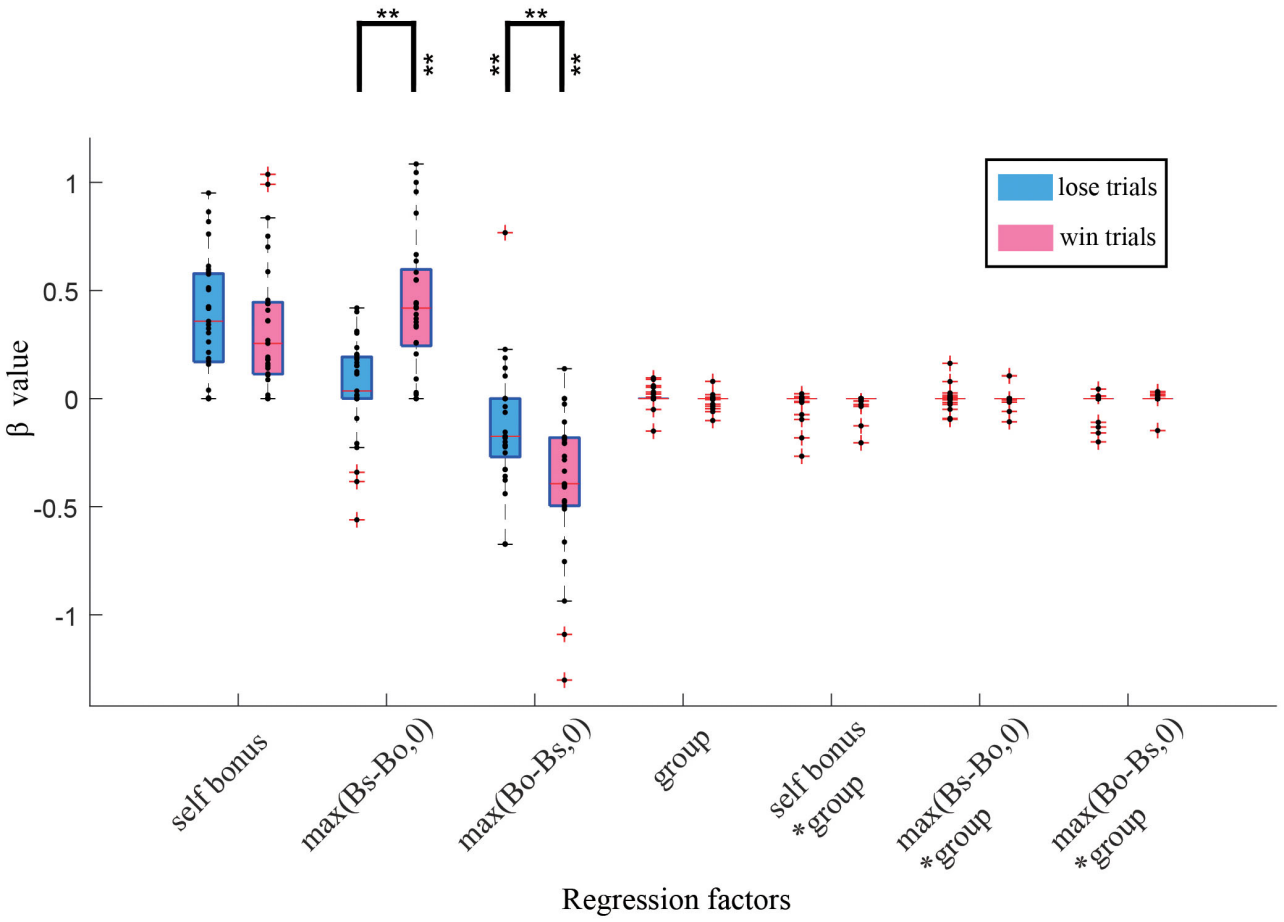
Boxplot of β values for winning trials and losing trials. A Lasso regression was conducted separately for win and loss trials. Blue and red represents lose and win trials, respectively. Participants showed significantly different β values for max (Bo-Bs,0) and max (Bs-Bo,0) between win and lose situations. **p < 0.01.

To examine how the outcome impacts participants' ratings more precisely, we conducted another LASSO regression that separated the win and loss trials (Figure 5). We found that both advantage [max (Bs-Bo,0)] and disadvantage max (Bo-Bs,0) had a larger (positive and negative, respectively) effect in loss trials than in win trials (*t*-test, *p* < 0.01) and that the effect of advantage in the loss trials was not significant. Interestingly, both win and loss trials showed a comparable β value for self-bonus. These findings clarified that outcome (win or loss) has a critical effect on the link between social inequity (i.e., advantage and disadvantage) and social valuation but not on the contribution of self-bonus.

Previous studies have demonstrated that the prediction error (PE) has a strong effect not only on decision making but also on wellbeing (Rutledge et al., 2014). Because PE may also influence social valuation in this study, we conducted Lasso and GLM analyses based on PE, where we used PE as a regressor instead of group and defined:


$$\begin{array}{l} PE_{n}=X_{n}-E, \end{array}$$

where *PE*_*n*_ represents the prediction error in trial n, *X*_*n*_ is the outcome (win/loss) of that trial, and E is the expectation of winning (win rate) in that trial. Since participants successfully learnt the social hierarchy in task 1, we used the fixed win rate (see Figure 1B) for each opponent.

We compared the effect sizes of outcome and PE (Supplementary Figure S3). When fed into a same regression, β values for outcome were significantly different from 0, indicating that participants cared more about outcome than PE.

We next investigated the effect of SDO on individual differences in social valuation by calculating the correlation coefficients between LASSO β values for each term and the total SDO score, SDO-D, and SDO-A (Table 1). We found a significant correlation for max (Bo-Bs,0)^*^outcome, which suggests that the individual differences in social valuation seen in Figures 4, 5 [i.e., for max (Bo-Bs, 0) and outcome] can be explained by SDO. In addition, we found significant correlations for Bs^*^group and max (Bs-Bo,0)^*^group.

**Table 1:** Correlation between LASSO β values and SDO scores.

**Personality traits**	**SDO-A**	**SDO-D**	**SDO-total**
**Factors**			
Bs	0.21	0.26	0.25
max(Bs-Bo, 0)	0.22	0.15	0.21
max(Bo-Bs, 0)	−0.11	0.03	−0.05
outcome	−0.17	−0.33	−0.26
group	−0.04	0.03	−0.01
Bs ^*^outcome	−0.03	−0.03	−0.03
max(Bs-Bo, 0) outcome	−0.27	−0.21	−0.27
max(Bo-Bs, 0) ^*^outcome	0.59^**^	0.47^*^	0.58^**^
Bs ^*^group	−0.39^*^	−0.13	−0.30
max(Bs-Bo, 0) ^*^group	0.27	0.42^*^	0.37
max(Bo-Bs, 0) ^*^group	−0.21	0.05	−0.11
outcome group	−0.08	−0.18	−0.14
constant			

^*^p < 00.050. Bs, self-bonus; Bo, opponent-bonus0.

**Figure 6:**
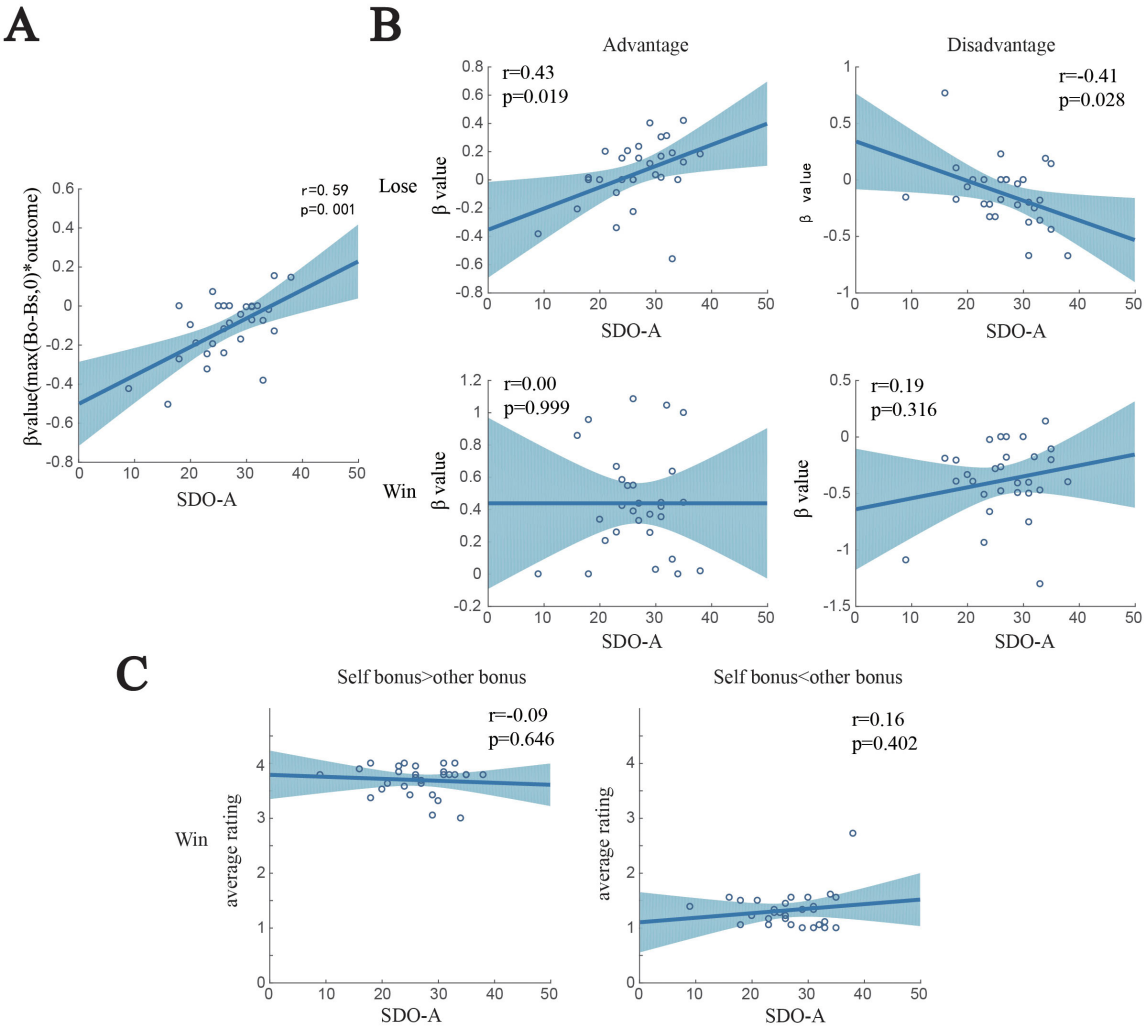
Correlation between β values, behavior data, and SDO-A. **(A)** β values for max (Bo-Bs,0)*outcome and SDO-A (*r* = 0.59, *p* = 0.001) are significantly correlated. **(B)** β values from the Lasso regression conducted separately for outcomes. In the loss trials, β values were correlated with the SDO-A score (top), but in the winning trials, there was correlation (bottom). **(C)** To see how wins impact the participants, we separated win trials into two types: one when participants get more reward than the opponent, and the other when the opponent gets more reward than the participants. The data shows winning has a great influence on all participants in that participants rate higher when they get more in winning trials.

**Figure 7:**
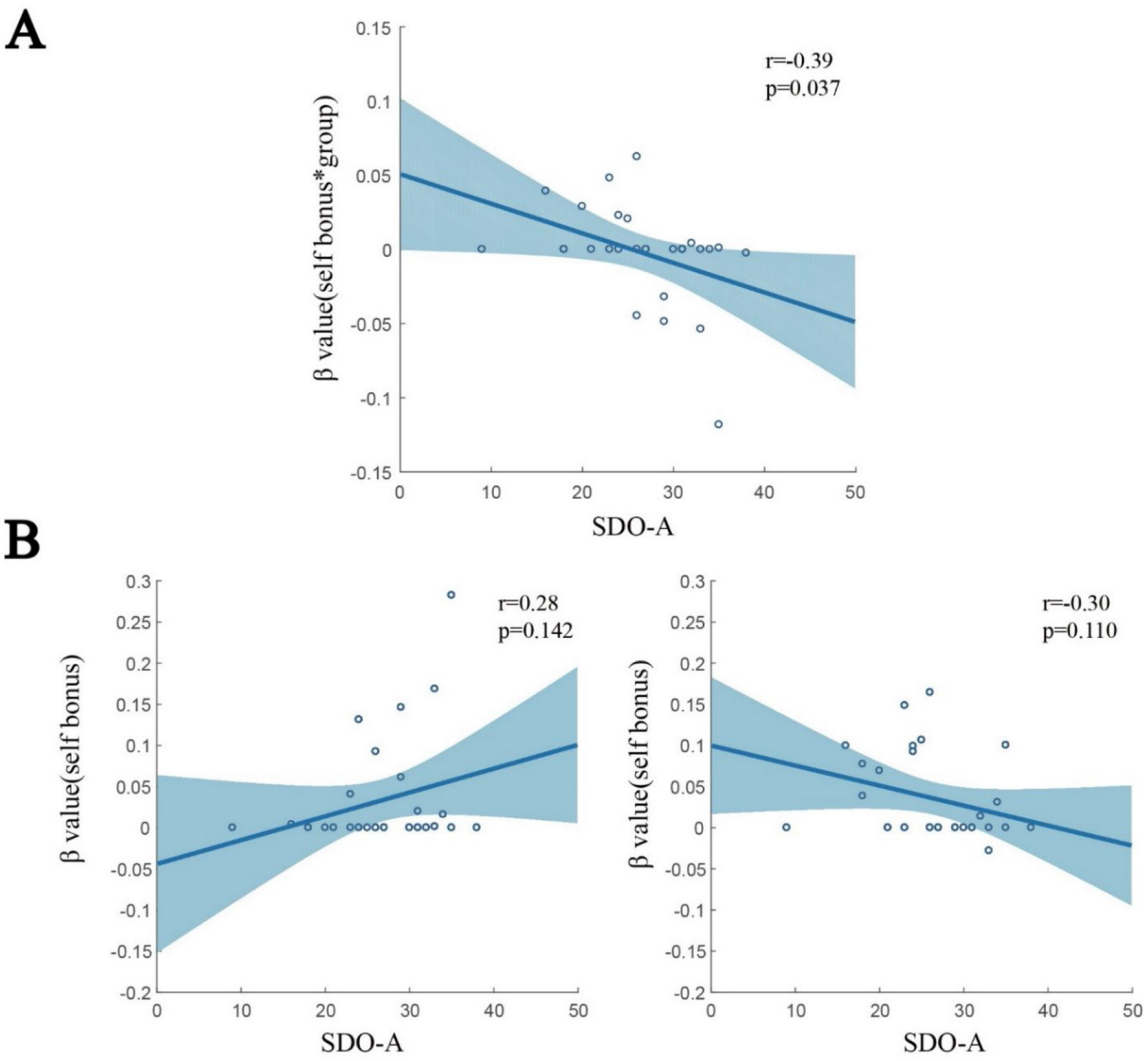
Correlation between LASSO β values, group, and SDO-A. **(A)** A significant correlation between β values for Bs*group and SDO-A was found (*r* = −0.39, *p* = 0.037). **(B)** When separating the trials for lower (left) and higher (right) groups, β values for each group tended to show an opposite correlation with the SDO-A score.

To understand what the correlations of SDO represent, we display the correlation of SDO-A with max (Bo-Bs,0)^*^outcome (Figure 6) and with Bs^*^group (Figure 7). In Figure 6A, the β value for max (Bo-Bs,0)^*^outcome correlates positively with SDO-A (*r* = 0.59, *p* = 0.001). This result indicates the relationship between SDO and social valuation to disadvantage depends on the outcome. Therefore, we separately conducted LASSO regressions for outcome (win/loss) and (dis)advantage and plotted the β values and SDO. We found that in the loss trials, β values for advantage and disadvantage significantly correlated with SDO-A (*r* = 0.43, *p* = 0.019, and *r* = 0.41, *p* = 0.028, respectively) but not in the win trials.

We further separated win trials into advantageous (self-bonus > other-bonus) and disadvantageous (other-bonus > self-bonus) situations and calculated the average rating in each situation (Figure 6C). We found that all participants respectively rated high and low in the advantageous and disadvantageous situations irrespective of SDO. These findings demonstrated that wins and losses critically affect our valuation process under the influence of SDO. More specifically, after winning a game, both low and high SDO-A individuals rated higher and lower in the advantageous and disadvantageous situation, respectively. By contrast, after losing a game, low SDO-A individuals rated higher in the disadvantageous situation, while high SDO-A individuals rated higher in the advantageous situation.

In addition to the LASSO regression, we conducted a GLM analysis using the same regressors (Supplementary Figure S1) and correlated the GLM β values with the SDO-A score (Supplementary Table S1 and Supplementary Figure S2). The results were highly consistent with the results of the LASSO regression.

Finally, we examined the effects of group and SDO on social valuation. As shown in Figure 7A, β values for Bs^*^group correlated negatively with SDO-A (*r* = −0.39, *p* = 0.037). This finding suggests that low and high SDO-A individuals rated lower and higher when they received more self-bonus against the lower group, respectively, but higher and lower against the higher group. To validate this finding, we separated the group variable into the lower (left) and higher (right) groups (Figure 7B). Indeed, we found the tendency that against lower-group opponents, low and high SDO-A individuals rated lower and higher when they obtained a higher self-bonus, respectively. By contrast, against higher-group opponents, low and high SDO-A individuals tended to rate higher and lower when they received a higher self-bonus, respectively. These results revealed that low and high SDO-A individuals do not and do like obtaining large bonuses when competing with a lower-group opponent, respectively.

In addition, we performed a similar correlation analysis between β values for PE and SDO (Supplementary Figure S4 and Supplementary Table S2). We found a negative correlation between PE^*^Bs and SDO-A, which illustrates SDO-A to a degree characterized the PE influence on social valuation, but only through self-reward and not inequity.

## 4 Discussion

In this study, we demonstrated that individual differences in attitudes toward social dominance and hierarchical structure, as measured by SDO, play important roles in the dynamic computation of social valuation through the interaction with the outcome (win or loss). More specifically, we found that wins and losses modulate the effect of social inequity on social valuation differently: in loss trials, both advantageous and disadvantageous bonus distributions had stronger influences on social valuation compared to win trials. We also observed that in loss trials, low-SDO individuals rated disadvantageous bonuses more favorably, whereas high-SDO individuals preferred advantageous ones. This pattern was reversed in win trials. Regarding the opponent's social rank, low-SDO participants rated scenarios more positively when a lower-ranked opponent received a higher bonus during loss trials, while high-SDO individuals consistently favored larger bonuses regardless of outcome or rank.

Previous research has shown that winning compared to losing positively contributes to subjective wellbeing in non-social contexts (Rutledge et al., 2014; Imas, 2016). These studies demonstrated that reward prediction error—a proxy for win/loss—has a greater impact on subjective wellbeing than either the chosen or expected reward. The present work extends these studies by exploring win/loss effects in a social context and by examining how these effects interact with social inequity, social rank, and SDO. Notably, we found that not only does win/loss interact with social inequity to shape social valuation, but they also interact with SDO, revealing a layered structure of social affective computation.

While previous studies have investigated the influence of SDO on decision making, most have used simple contexts or hypothetical scenarios. For example, low-SDO individuals tend to exhibit greater empathy toward others in pain (Chiao et al., 2009). In contrast, high-SDO individuals have been shown to make more unethical choices in leadership simulations (Son Hing et al., 2007). However, the effect of SDO on evaluations in complex, competitive contexts has remained largely unexplored.

Neuroscientific studies have also begun to uncover the neural substrates of dominance hierarchy perception. For example, the rostro-medial prefrontal cortex has been shown to track dominance-related hierarchy in competitive games (Ligneul et al., 2016). Other work has demonstrated that the amygdala, hippocampus, and ventromedial prefrontal cortex track perceived social hierarchies, while the medial prefrontal cortex specifically tracks hierarchies in which the individual is involved (Kumaran et al., 2016). Furthermore, SDO has been linked to individual variability in rank sensitivity in the right anterior dorsolateral prefrontal cortex (Ligneul et al., 2017). Nevertheless, few studies have examined how the SDO contributes to individual differences in social valuation.

It is notable that exploitation in our task was tied to two components: the outcome of the competitive game and the social status of the opponent. We initially hypothesized that when the winner received a higher bonus than the loser, the participants would perceive this outcome as a form of justified exploitation—one that high-SDO individuals would especially value. Interestingly, however, our results revealed that when participants won, low-SDO individuals expressed dissatisfaction if they received less than their opponent. Post-experiment interviews suggested that some of them were angered when they won and received less.

Further behavioral considerations support these observations: low-SDO individuals did not appear to view receiving a higher bonus after a win as an exploitation of their advantage but rather as a fair reward for effort. For them, an imbalance—such as a winner receiving less than the opponent—was perceived as unfair regardless of who it applied to. In contrast, high-SDO individuals were dissatisfied with lower bonuses across both win and loss conditions. They appeared to view receiving more than others as justified and were indifferent to fairness concerns. This pattern is consistent with previous research showing a link between SDO and Competitive Jungle (CJ) belief, i.e., the notion that society is a ruthless competition where stronger individuals deserve more (Chirumbolo et al., 2016).

Our data also suggested that participants, especially those low in SDO, considered “winners receiving more” as fair and reasonable. From a sociological perspective, social hierarchy has been conceptualized along two dimensions: dominance (imposed power) and prestige (earned respect through competence). Even in egalitarian societies such as hunter-gatherer communities, rank differences arise naturally based on perceived success (Garfield and Hagen, 2020), and individuals tend to associate fairness with earnings proportional to effort (Fehr and Schmidt, 1999). This tight coupling between social grades in our task and real society suggests a possibility that our grade ratings learned in the competitive task share key characteristics with the real social grade. However, it is necessary to validate this view further in future studies.

We observed that large individual differences mainly emerged when participants lost. One possible interpretation for this observation is that in win trials, high-SDO participants' desire for dominance was temporarily satisfied, aligning with the fairness of the situation. However, in loss trials, their intrinsic preference for self-advantage re-emerged, making them favor inequitable distributions. In contrast, low-SDO individuals rated trials more positively when the opponent received more, interpreting the result as fair given the opponent's effort. In essence, high-SDO individuals appear to prioritize self-interest, while low-SDO individuals prioritize fairness. This view is consistent with previous studies. Karunaratne's group, for example, reported that high SDO individuals usually refuse to apologize to another group to maintain their dominant position (Karunaratne and Laham, 2019). Other studies also suggested that high SDO individuals have high authoritarianism tendency (Passini, 2008) and more racism (Caviola et al., 2019). However, the desire-for-dominance hypothesis of high-SDO participants needs further empirical support.

This study provides important implications for real-life situations involving social comparison, such as the design of incentive policies. We observed that individuals with low social SDO tended to perceive it as reasonable for winners to receive greater rewards, while those with high SDO prioritized their own rewards. To maintain motivation across different individuals, it may be effective to adopt a performance-based distribution system that also links team performance to individual compensation. In line with this, when forming teams for competitive contexts, it might be beneficial to place high-SDO individuals in teams that regularly achieve rewards, while low-SDO individuals may prefer teams that emphasize fairness.

This study has several limitations. First, although we found that low-SDO participants rated trials lower when they received more than a lower-status opponent, which appears intuitive, we found that the LASSO β coefficients for group-related terms were relatively low. This result may be attributable to the task design, which did not make the group membership serve as a salient cue. Second, the task could be simplified in future studies. For instance, statistical results may become more robust by reducing the number of opponents in the learning task and making the number of trials in different conditions more comparable. Finally, the sample size of the present study was limited. Although we conducted a power analysis that concluded the current sample size is sufficient for our analysis and several other studies have been based on similar sample and effect sizes (Blagrove and Akehurst, 2001, Smillie et al., 2011), it is desirable that future studies will replicate the results of this study by using larger numbers of participants.

Finally, there are several important directions for future research. First, it is crucial to ensure that participants can learn the social dominance hierarchy more easily, which may also facilitate collecting larger samples. One potential approach is to reduce the number of opponents in the task. Additionally, identifying the neural substrates underlying the context-dependent social valuation examined in this study would be valuable. This could be achieved by conducting neuroimaging experiments using the same social valuation task. Furthermore, testing a larger and more diverse sample, including participants from different age groups and cultural backgrounds, is important for establishing the robustness and generalizability of the observed effects.

## Data Availability

The raw data supporting the conclusions of this article will be made available by the authors, without undue reservation.
