# Supplementary material for: Social dominance orientation underlies social valuation in a competitive social hierarchy
Source: Front Psychol. 2025 Sep 15;16:1615364. doi: 10.3389/fpsyg.2025.1615364 (PMC12477826; doi:10.3389/fpsyg.2025.1615364)
Supplement: Supplementary file 1 [file Supplementary_file_1.docx]

**Supplementary Information**


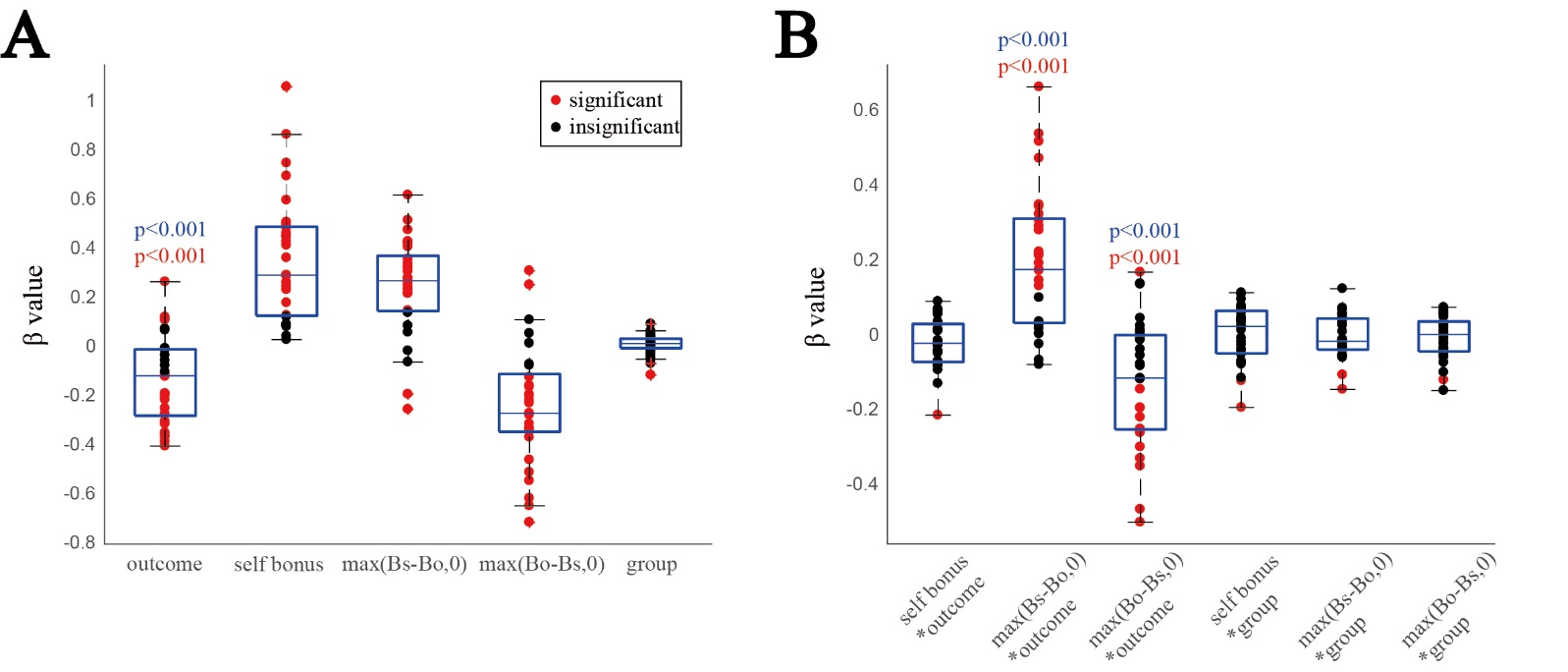


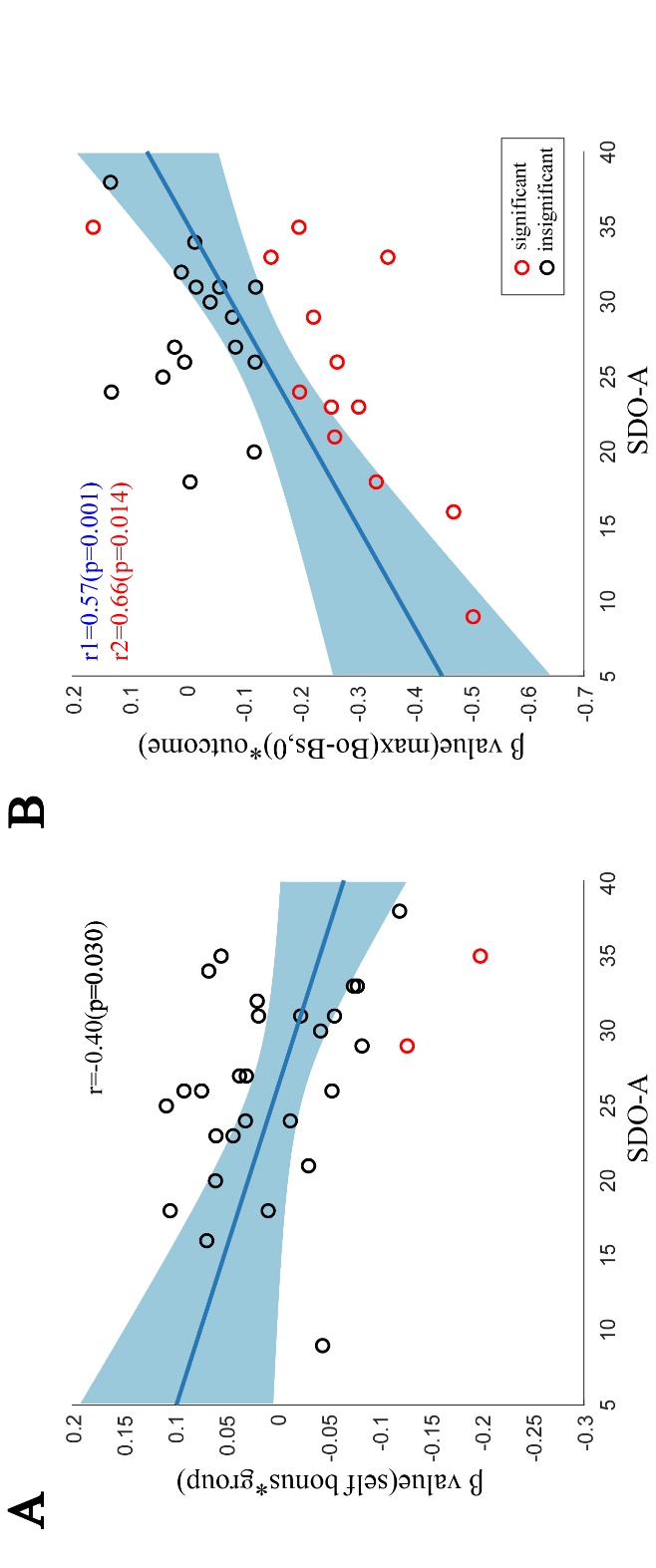


**Fig. S1 Boxplot of β values for 5 factors (A) and their interaction terms (B).** R^2^>0.7 was used as the criterion to select the participants well-described by the GLM. Red points show significant individuals and black points show insignificant individuals.

**Fig. S2 Correlation between SDO-A and β values for Bs*group (A) and max(Bo-Bs,0)*outcome (B).**

R^2^>0.7 was used as the criterion to select the participants well-described by the GLM. Red points show significant individuals and black points show insignificant individuals.

**Table S1 Correlation between β values and SDO scores.**

|  | SDO-A | SDO-D | SDO-total |
| --- | --- | --- | --- |
| constant | -.13 | -.22 | -.18 |
| Bs | .20 | .25 | .24 |
| max(Bs-Bo,0) | .23 | .18 | .23 |
| max(Bo-Bs,0) | -.08 | .06 | -.02 |
| outcome | -.17 | -.27 | -.23 |
| group | .30 | .05 | .20 |
| Bs *outcome | .00 | .06 | .03 |
| max(Bs-Bo,0) *outcome | -.26 | -.19 | -.25 |
| max(Bo-Bs,0) *outcome | .54* | .43* | .53* |
| Bs *group | -.40* | -.21 | -.35 |
| max(Bs-Bo,0) *group | .30 | .49* | .41* |
| max(Bo-Bs,0) *group | -.24 | -.02 | -.15 |
| group *outcome | -.05 | -.08 | -.07 |

*p<0.05. Bs, self-bonus; Bo, opponent-bonus.


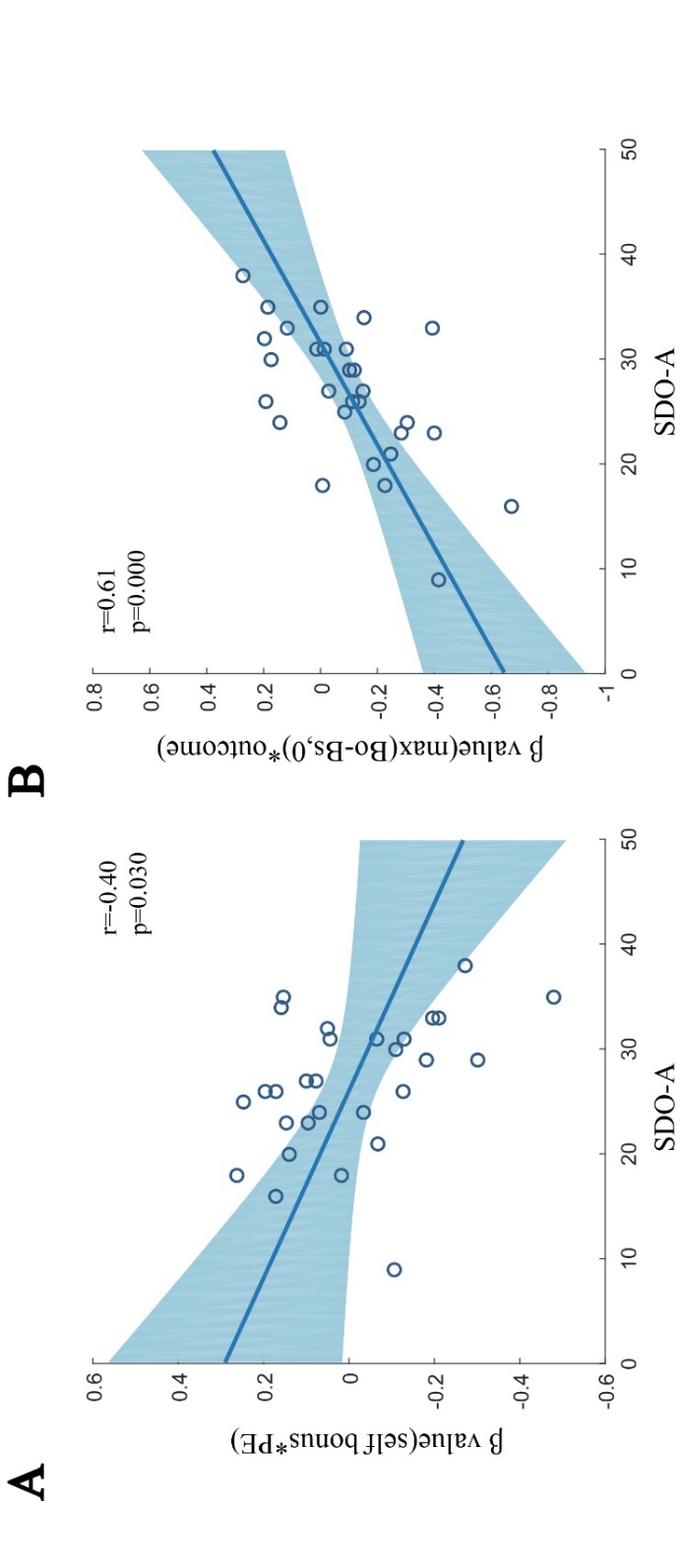

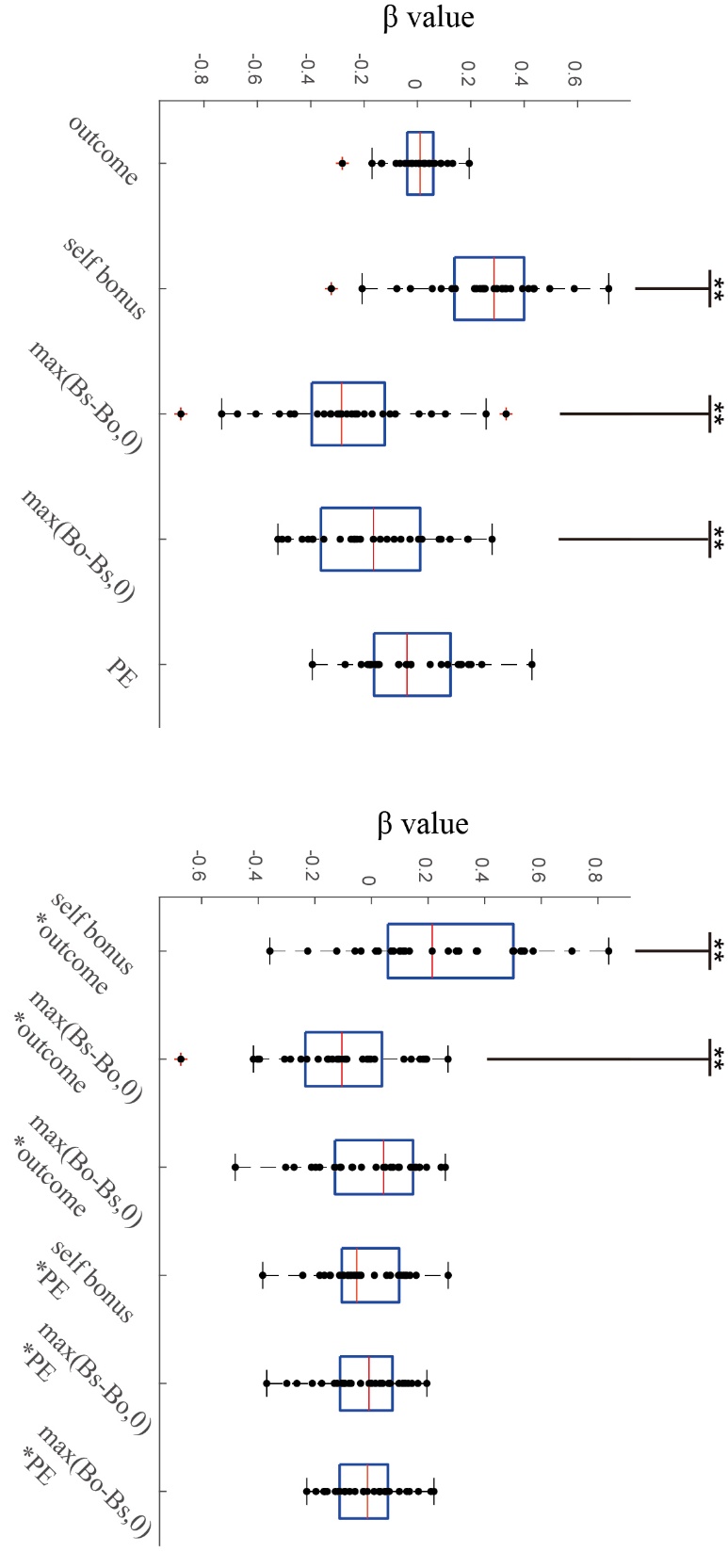


**Fig. S3 β values for Lasso regression with the prediction error (PE).**

*p<0.05, **p<0.01. PE: prediction error.

**Fig. S4 Correlation between SDO-A and β values for Bs*PE (A) and max(Bo-Bs,0)*outcome (B).**

PE: prediction error.

**Table S2 Correlation between β values and SDO scores.**

|  | SDO-A | SDO-D | SDO-total |
| --- | --- | --- | --- |
| Bs | .20 | .25 | .24 |
| max(Bs-Bo,0) | .23 | .18 | .23 |
| max(Bo-Bs,0) | -.08 | .06 | -.02 |
| outcome | -.09 | -.29 | -.19 |
| PE | -.05 | .08 | .00 |
| Bs *outcome | .43* | .25 | .38 |
| max(Bs-Bo,0) *outcome | -.34 | -.39 | -.39 |
| max(Bo-Bs,0) *outcome | .61** | .38 | .55 |
| Bs * PE | -.40* | -.21 | -.35 |
| max(Bs-Bo,0) * PE | .30 | .49 | .41 |
| max(Bo-Bs,0) * PE | -.23 | -.02 | -.15 |
| PE *outcome | -.05 | -.08 | -.07 |

*p<0.05, **p<0.01. Bs, self-bonus; Bo, opponent-bonus; PE, prediction error.
